# Quercetin inhibits degranulation and superoxide generation in PMA stimulated neutrophils

**DOI:** 10.2478/v10102-012-0014-5

**Published:** 2012-06

**Authors:** Jana Pečivová, Tatiana MačIčková, Klára Sviteková, Radomír Nosáľ

**Affiliations:** 1 Institute of Experimental Pharmacology & Toxicology, Slovak Academy of Sciences, SK-84104 Bratislava, Slovakia; 2 National Transfusion Service, Bratislava, Slovak Republic

**Keywords:** neutrophils, quercetin, superoxide generation, myeloperoxidase

## Abstract

Activated neutrophils represent the main source of myeloperoxidase (MPO), superoxide (SO) and subsequently derived oxygen metabolites. They have important microbicidal activities, however in inflammatory conditions they may secondarily attack surrounding tissues. Overproduction of reactive oxygen species, prolonged or excessive liberation of MPO and other effective yet also toxic substances from neutrophils may participate in disturbed apoptosis, intensify the inflammatory processes and result in serious human diseases. The inhibitory effect of quercetin on PMA stimulated SO generation in isolated human neutrophils was found to be dose-dependent, without affecting the activity of intact isolated neutrophils. At comparable conditions, quercetin was more potent in inhibiting MPO release than SO generation. Our results indicate that quercetin could support resolution of inflammation through decreased activity of neutrophils, *i.e.* respiratory burst and degranulation.

## Introduction

Quercetin belongs to the group of flavonoids, natural compounds, produced as plant secondary metabolites with an important role in biological processes, such as pigmentation, germination, pollination attraction, and UV light protection (Rackova *et al.*, 2005).

Flavonoids are a group of phenolic compounds widely occurring in the plant kingdom, which exhibit a reported strong antioxidant capacity along with many other biological activities (interaction with protein phosphorylation and iron chelating (Bonina *et al.*, 1996).They may regenerate other antioxidants, by donating a hydrogen atom to the radical (Boyle *et al.*, 2000). Consumption of plant phenolics may be associated with a decreased risk of cardiovascular disease by stabilizing and protecting vascular endothelial cells against oxidative and pro-inflammatory insults (Reiterer *et al.*, 2004). Quercetin interferes with the proinflammatory signaling of thrombin resulting in the inhibition of adenosine nucleotide secretion from activated platelets and decreased neutrophil function (Kaneider *et al.,* 2004). Further flavonoids are potential agents that could reduce the risk of cataract formation (Stefek, 2011).

**Figure 1:**
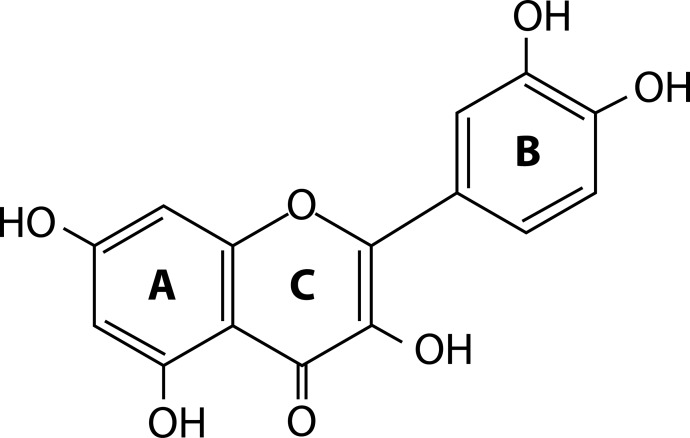
Molecular structure of quercetin.

Quercetin (Figure 1) is considered to be a very effective antioxidant and free radical scavenger due to some specific 1/ structural, 2/ molecular and 3/ conformational characteristics, such as the presence of a catechol moiety in the B ring and a 2,3-double bond in the C ring (Bors & Michel., 2002).

Liu *et al.* (2005) have sugested that the inhibitory effect of quercetin on stimulus-induced superoxide generation in human neutrophils is connected with parallel suppression of PMA-induced serine/threonine phosphorylation and the translocation of the cytosolic proteins p47^phox^ and p67^phox^ to the cell membrane.

The aim of this study was to assess the effect of quercetin on PMA stimulated SO generation in isolated human neutrophils and their degranulation measured as MPO release.

## Methods

Quercetin and phorbol-myristate-acetate (PMA) were obtained from Sigma, Germany.

Effect of quercetin on the activity of PMA stimulated neutrophils was evaluated on the basis of superoxide generation and MPO release, measured spectrophotometrically as superoxide dismutase inhibitable reduction of cytochrome c (Pecivova *et al.*, 2007) and *o-*dianizidine reduction in the presence of hydrogen peroxide (Somersalo *et al.*, 1990 ), respectively.

### Statistical analysis

All values are given as means ± SEM. The statistical significance of differences between means was established by Student's t-test and *p*-values below 0.05 were considered statistically significant.

## Results

**Figure 2:**
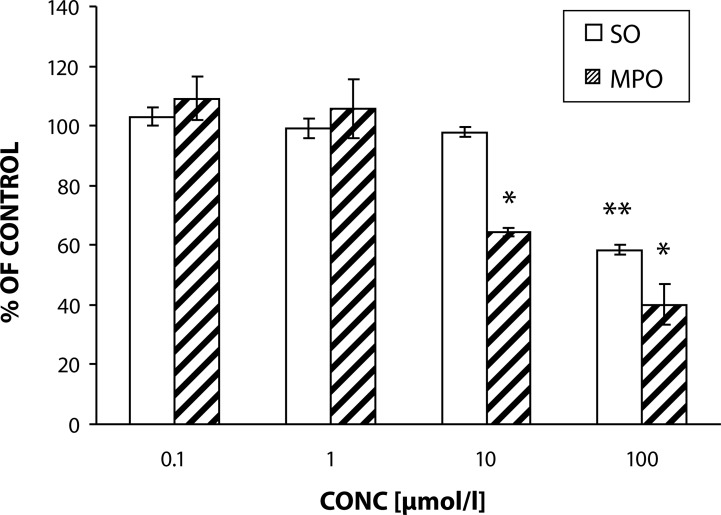
Effect of quercetin on PMA (1µmol/l, 15 min/37°C) stimulated superoxide generation in and MPO release from isolated human neutrophils. Results are the mean ± SEM, n=6, expressed as percentage of absolute average control value (PMA) of superoxide generation and MPO release: 50.41 ± 2.14 nmoles/10^6^ neutrophils/min and 3.09 ± 0.29 ΔA/Δt (calculated as area under curve), respectively.

Effect of quercetin in concentration 0.1–100 µmol/l on PMA stimulated SO generation in and MPO release from isolated human neutrophils was dose dependent (Figure 2). Resting cells were not affected by quercetin.

## Discussion

The antioxidant capacity of flavonoids is conferred by a high number of hydroxyl substitutions in each flavonoid molecule, which has a direct effect on the donating ability of hydrogen atoms to scavenge free radicals (Pietta, 2000).

Due to the unique chemical structure of quercetin (high number of hydroxyl substituents, C-2,3 double bond), it has been reported to be an effective scavenger of peroxyl radicals and nitric oxide in chemical systems, and to decrease dose dependently ROS and NO production in LPS-stimulated RAW 264.7 macrophages (Číž *et al.*, 2008).

In our experiments the incubation of isolated human neutrophils with quercetin diminished their PMA-activated production of ROS and MPO release in the highest concentration [100 µmol/l] by 42% and 60%, respectively. The concentration of 10 µmol/l significantly inhibited only MPO release by 36%. Our results are in agreement with findings of Nosal *et al.*, (2011) and Perečko *et al.* (2008) where quercetin dose dependently decreased chemiluminescence of whole blood and extra- as well as intra-cellular ROS generation of isolated neutrophils.

Quercetin is believed to protect human organism against several degenerative diseases (Hollman & Katan, 1997; Murota & Terao, 2003). The results indicate that the mechanism of the anti-inflammatory effect of quercetin involves interaction with NADPH oxidase (Liu *et al.*, 2005), iNOS protein expression (Číž *et al.*, 2008) and prevention of lipid peroxidation (Kotyzova *et al.*, 2007). Quercetin further modulates calcium homeostasis and cell signalling via Ca^2+^-ATPase (Horakova, 2011), and scavenges generated ROS (Číž *et al.*, 2008). Quercetin was found to inhibit both acute and chronic phases of experimental model of inflammation (Guardia *et al.,* 2001).

Our study provided evidence supporting the potential beneficial effect of quercetin in diminishing tissue damage at the site of inflammation by inhibiting MPO release and by decreasing the generation of superoxide and the subsequently derived ROS.

## References

[1] Bonina F, Lanza M, Montenegro L, Puglisi C, Tomaino A, Trombetta D, Castelli F, Saija A (1996). Flavonoids as potential protective agents against photo-oxidative skin damage. Int J Pharm.

[2] Bors W, Michel C (2002). Chemistry of the antioxidant. Effect of polyphenols. Ann NY Acad Sci.

[3] Boyle SP, Dobson VL, Duthie SJ, Hinselwood DC, Kyle JAM, Collins AR (2000). Bioavailability and efficiency of rutin as an antioxidant: A human supplementation study. Eur J Clin Nutr.

[4] Číž M, Pavelková L, Gallová L, Králová J, Kubala L, Lojek A (2008). The influence of wine polyphenols on reactive oxygen and nitrogen species production by murine macrophages RAW 264.7. Physiol Res.

[5] Guardia T, Rotelli AE, Juarez AO, Pelzer LE (2001). Anti-inflammatory properties of plant flavonoids. Effects of rutin, quercetin and hesperidin on adjuvant arthritis in rat. Il Farmaco.

[6] Hollman PCH, Katan MB (1997). Absorption, metabolism and health effects of dietary flavonoids in man. Biomed & Pharmacother.

[7] Horáková L (2011). Flavonoids in prevention of diseases with respect to modulation of Ca-pump function. Interdiscip Toxicol.

[8] Kaneider NC, Mosheimer B, Reinisch N, Patsch JR, Wiedermann CJ (2004). Inhibition of thrombin-induced signaling by resveratrol and quercetin: effects on adenosine nucleotide metabolism in endothelial cells and platelet-neutrophil interactions. Thromb Res.

[9] Kotyzova D, Černá P, Koutenský J, Hes O, Eybl V (2007). Effect of curcumin and quercetin on oxidative tissue damane induced by ferric nitrilotriacetate (Fe-NTA). Chem Listy.

[10] Liu G, Wang W, Masuoka N, Isobe T, Yamashita K, Manabe M, Kodama H (2005). Effect of three flavonoids isolated from japanese polygonum species on superoxide generation in human neutrophils. Planta Med.

[11] Murota K, Terao J (2003). Antioxidative flavonoid quercetin: implications of its intenstinal absorption and metabolism. Arch Biochem Biophys.

[12] Nosal R, Drábková K, Harmatha J, Jančinová V, Mačičková T, Pečivová J, Perečko T (2011). On the pharmacology of oxidative burst of neutrophils. Interdiscip Toxicol.

[13] Pečivová J, Mačičková T, Lojek A, Gallová L, Číž M, Nosál R, Holomáňová D (2007). In vitro effect of carvedilol on professional phagocytes. Pharmacol..

[14] Perečko T (2008). Effect of the extract from Arctostaphylos uva-ursi L. On the chemiluminescence of whole blood and isolated neutrophils. Pokroky vo farmakológii vSlovenskej Republike III. PEEM, Bratislava.

[15] Pieta PG (2000). Flavonoids as antioxidants. J Nat Prod.

[16] Rackova L, Firakova S, Kostalova D, Stefek M, Sturdik E, Majekova M (2005). Oxidation of liposomal membrane suppressed by flavonoids: Quantitative structure–activity relationship. Bioorg Med Chem.

[17] Reiterer G, Toborek M, Hennig B (2004). Quercetin protects against linoleic acid-induced porcine endothelial cell dysfunction. J Nutr.

[18] Somersalo K, Salo OP, Bjorksten F, Mustakalio KK (1990). A simplified Boyden chamber assay for neutrophil chemotaxis based on quantitation of myeloperoxidase. Anal Biochem.

[19] Stefek M (2011). Natural flavonoids as potential multifunctional agents in prevention of diabetic cataract. Interdiscip Toxicol..

